# Introduction to Ethics and Global Health

**DOI:** 10.1186/s12910-018-0278-1

**Published:** 2018-06-15

**Authors:** Beatrice Godard, Slim Haddad, Robert Huish, Daniel Weinstock

**Affiliations:** 1 Department of Social and Preventive Medicine, École de santé publique, Université de Montréal, PO Box 6128, Station Centre-ville, Montreal, H3C 3J7 Canada; 2 Department of Social and Preventive Medicine, Université Laval, Pavillon Ferdinand-Vandry, 1050, avenue de la Médecine, Québec, Québec G1V 0A6 Canada; 3 Department of International Development Studies, Dalhousie University, 6135 University Avenue, Room 3038, Halifax, NS B3H 4R2 Canada; 4 McGill Institute for Health and Social Policy, New Chancellor Day Hall, 3644 Peel Street Montreal, Quebec, H3A 1W9 Canada

This supplement, *Ethics and Global Health*, is the result of a joint initiative of young researchers and mentors engaged in a training program called the *Global Health Research Capacity Strengthening Program* (GHR-CAPS). Mentors of this initiative contribute to the national and international development of global health research by training researchers who will work in a high-calibre interdisciplinary environment and whose research will influence policy. GHR-CAPS provides an inter-university platform for teaching and training in global health that includes courses, seminars, short-term internships, and an annual summer school. Between 2009 and 2016, the program hosted about 51 junior researchers from all over the world. They were encouraged to engage actively in the GHR-CAPS community and the production of collaborative scientific work. In 2016, the summer school was transformed into a winter school and focused on the theme *Ethics and Global Health*. More specifically, the goal was to highlight some of the ethical challenges doctoral and postdoctoral fellows encountered in their research experiences, whether in designing, implementing, or disseminating the results of their studies. Fellows were encouraged to engage in collective reflection on ethical challenges in global health, which could be informed by their own experience as a young researcher, or by ethical issues related to global health interventions. As such, this supplement’s theme is varied and covers a broad spectrum of topics in relation to research or intervention in global health. The range of themes shows that the ethical challenges in global health are diverse and, moreover, that they are ever-changing.

Ethical debates constantly emerge because of the contexts in which they occur: population frailty or vulnerability, resource constraints, lack of human rights protection, crisis or pandemic, etc. Given their variety, there is no single way to address ethical challenges in global health. For the authors in this supplement, addressing them could involve self-reflection on their own approach to research, or reflecting more broadly on ethical issues that have arisen during their research processes or that were identified from the results of their research.

We welcomed both empirical research reports and reflective, critical contributions that deepen the ethical understanding of global health, whether in relation to the nature of global health, or global health research or professional interventions. The supplement also includes critical reflections on global health and conventional ethics: the foundations of ethics; normative or empirical approaches to ethics, and how Western or non-Western approaches to ethics may be more or less appropriate in global health. This is to say, our supplement looks at the limitations of, and contributions to, research and action in global health that can benefit individuals and communities.

Ten fellows joined their efforts to reflect on issues related to Ethics and Global Health. With the support of four senior mentors from the Program who are the editors of this special issue, they have prepared a collection of articles that discuss the ethical challenges inherent to global health. As expected, the collection covers a broad spectrum of topics and ethical issues that can be grouped into three thematic groups: 1) practices of researchers; 2) institutions and Institutional Revision boards (IRBs) roles; and 3) citizens’ and communities’ rights during interventions.

Regarding the first group, the contributions address some of the ethical dilemmas the fellows faced in their research projects. Turcotte-Tremblay and Mc Sween-Cadieux present how it can be a challenge for researchers to protect confidentiality of participants while locally disseminating results of health systems research to stakeholders. The latter may be able to identify research participants, or at least groups of participants, especially in rural healthcare centres that tend to be small settings. Indeed, some individuals have unique positions in the healthcare system that make them more easily identifiable by local stakeholders familiar with the environment. While identifying a number of potential “strategies that can help researchers minimize this difficulty and improve ethical research practices”, the authors consider that “reflections surrounding ethical issues in global health research should be deepened to better address how to manage competing ethical responsibilities while promoting valuable research uptake.” Another ethical challenge commonly encountered in research practices of global health researchers relates to an equal positioning of Northern and Southern researchers. Taking the example of partnerships between Western and African researchers, Gautier shows that the creation in the 1980s of “a scientific hub of working relationships based on material differences, in a context that could not but lead to tensions between the alleged ‘partners’,” mostly due to the maintenance of paternalistic approaches by Northern researchers. Following an analysis of the origins of global health partnerships, the author calls for a reconceptualization of “global health as an academic discipline, mainly by being explicit about past and present inequalities between Northern and Southern universities that this discipline has, so far, ignored.”

Furthermore, despite the development of ethical guidelines for global health research, some discrepancies often remain between what these guidelines recommend and their operationalization in the field. This is seen in two papers, one showing the limits of the role of the IRBs to prioritize a rule-based framework for guaranteeing researchers’ ethical behaviour (Daku), the other mapping out “the ethical tensions likely to arise in global health fieldwork as researchers negotiate the challenges of balancing ethics committees’ rules and bureaucracies with actual fieldwork processes in local contexts” (D’souza). At the intersection of research practices and the role of IRBs, Daku’s perspective is that “while appropriate for most situations, rule-based approaches tend to fall apart when the researcher is engaged in ‘ethically dangerous’ research.” Drawing on a recent ethnographic research project about drinking and driving in South Africa, Daku argues that “a rule-based framework is not always desirable, possible, effective, or consistent” and that “we need to go beyond the rules and regulations articulated by ethics boards and to focus more specifically on creating and nurturing virtuous researchers.” As for D’souza, drawing from an implementation and evaluation project in Jamaica, her view is that “global health research ethics should be premised not upon passive accordance with existing guidelines on ethical conduct, but on tactile modes of knowing that rely upon being engaged with, and responsive to, research participants.”

Community involvement in research through Community Advisory Boards (CABs) is another way of operationalizing ethical research (Fregonese). Taking the example of Community Based Participatory Research conducted with indigenous populations, Fregonese examines the role of community engagement in clinical research and observational studies on malaria. Like IRBs, CABs are not without limitations but the author sees them as “a model to import into clinical trials and observational research where no alternative model of community representation is currently being used. Allocating more resources to training and shifting more power to community representatives could be part of the solution to current CAB limitations.”

In another vein, by strictly considering the role of the IRBs and the institutions, Sambieni’s paper examines the differences and structural weaknesses of IRBs in four countries in the field of reproductive health. As stated by the author, “regardless of national contexts, the institutions responsible for research ethics, founded on international regulations, are all expected to be structured and to operate in a common way.” However, despite having the same mandate, his comparative study illustrates how IRBs examining the same project function differently, while they all exhibit the same weaknesses. It appears that IRBs demonstrate “the profound influence of context on the ways in which different institutions function and enforce regulations.” This may, however, be inevitable because international regulations have limitations.

Finally, when it comes to the interventions and rights of citizens and communities, “although many public policies are directed towards equity and protecting people’s rights, these are not comprehensively and inclusively applied in ways that prioritize the health rights of citizens” (Shafique et al.). That is what Shafique and her colleagues concluded about Bangladesh’s health system, given the rural–urban divide and the lack of coordination among implementing agencies. According to the authors, “the unregulated profusion of the private sector and immoral practices of service providers result in high out-of-pocket expenditures for the urban poor, leading to debt and further impoverishment.” They recommend that “state and non-state actors work together, understanding and acknowledging their moral responsibilities for improving the health of the urban poor by engaging multiple sectors.” This is also the case as reported by Mouliom, who highlights the unequal access to care in public maternity services in Cameroon. According to that author, “since the mid-1980s, there has been a gradual drift in the provision of maternal care … in Cameroon in particular despite the efforts put in place by government authorities.” As the author says, here “it is the patient’s economic capital that counts” and “as a result, to access these health services many women, particularly those who are financially vulnerable, experience a lot of difficulties.”

The lack of ethical considerations does not only concern interventions among underserved individuals or communities. Salwa and Al-Munim have monitored a human papillomavirus vaccination program targeting both grade-5 female students and girls not in school (ages 10–12 years) in Bangladesh. They identified several ethical problems, such as incomplete information about Human papillomavirus (HPV) and cervical cancer, lack of awareness among the implementers regarding ethical issues around HPV vaccination, lack of informed choice for both parents and girls, and the absence of follow-up plans for adverse effects in the long run. The authors also criticize the female-only strategy of the program and advocate that adolescent boys should be given HPV-related health education as well. It appears that “more ethical discussion and debate are needed among public health professionals in Bangladesh to increase awareness about ethical issues related to human health.” Indeed, integrating primary health care in low- and middle-income countries presents several challenges. The three previous papers demonstrate this.

For Druetz, at least two challenges must be considered: “1) to find and develop a “cross-cutting approach” [to implementation] that is operational and effective, and 2) to coordinate efforts in the health sector to overcome the tension between national programs and local NGO-driven interventions.” He develops his argument by drawing on observations made and lessons learned during a six-year research project evaluating the effects of interventions against malaria in Burkina Faso. Integration should be thought of as a process to reconcile two tensions according to Druetz, that is, “between selective versus comprehensive approaches, or fragmentation versus cohesion.” The author argues that “in the context that characterizes many LMICs today, better aid coordination and the strengthening of public health systems—as multisectoral approaches try to promote—might be among the best options to integrate primary healthcare interventions sustainably and ethically.”

Reading the ten articles that form this supplement, we could suggest that these challenges may not be new, but they are very real! Not only do such challenges hinder research and interventions in global health, but this review shows there are still many ethical challenges to overcome, despite the normative and operational frameworks available. Whether based on research practices (empirical or theoretical) or interventions (multidisciplinary, multicultural, or international), the experiences and reflections shared here reaffirm the importance of continuing the discussions initiated by many scholars to make research and interventions in global health more ethical. Part of this challenge is due to the wide scope of themes, issues, and challenges that global health addresses. It is difficult to effectively delineate a particular framework for ethics in global health research. The interest of these experiences and reflections is that they all constitute “ethics in action” exercises, with a view towards doing research better. They represent, in this sense, a great contribution to a better understanding of ethical issues specific to global health.

## Abbreviations

CABs: Community Advisory Boards
GHR-CAPS: Global Health Research Capacity Strengthening Program
HPV: Human papillomavirus
IRBs: Institutional Review Boards (or ethic committees)
LMICs: Low- and middle-income countries
NGO: Non-governmental organizations

